# *Epilepsia Open*—October 2025 Announcements

**DOI:** 10.1002/epi4.70151

**Published:** 2025-11-17

## ILAE CONGRESSES

Genetic Basis of Developmental and Epileptic Encephalopathies Workshop for the Eastern Mediterranean Epilepsy Health Care Providers

October 1–2, 2025

Sfax, Tunisia

Curso virtual sobre epilepsia en atención primaria para América Latina 2025‐2

6 October ‐ 30 November 2025

Visiting Teacher Program—Tunisia

November 6–8, 2025

Sfax, Tunisia

Cápsula virtual sobre terapia cetógena en epilepsia farmacoresistente 2025

3 ‐ 10 November 2025

Regional Training Course on Epilepsy

13 ‐ 14 November 2025

Conakry, Guinea

Visiting Teacher Program

November 13–15, 2025

Rabat, Morocco

Epilepsy in the Elderly: At the Crossroads of Brain Aging, Neurodegeneration, and Functional Networks

November 21–22, 2025

Marrakech, Morocco

**2026**

15th ILAE School on Pre‐Surgical Evaluation for Epilepsy and Epilepsy Surgery

January 19–23, 2026

Brno, Czech Republic

19th Latin American Summer School on Epilepsy

February 23–28, 2026

Guadalajara, Mexico

8th ILAE School on EEG in the First Year of Life

March 30, 2026 to April 2, 2026

Cambridge, UK

5th International Summer School of Neuropsychology

April 19–24, 2026

Picardy, France

ILAE School on Neuroimaging 2026

6 ‐ 9 May 2026

Potsdam, Germany

XIV Congreso Latinoamericano de Epilepsia

May 16–18, 2026

Lima, Peru

1°Curso Latinoamericano de EEG Pediátrico

August 6–9, 2026

TBC

16th European Epilepsy Congress

September 5–9, 2026

Athens, Greece

16th International Summer School for Neuropathology and Epilepsy Surgery

10 ‐ 13 September 2026

Erlangen, Germany

16th Asian & Oceanian Epilepsy Congress

November 19–22, 2026

Kuala Lumpur, Malaysia

**2027**

37th International Epilepsy Congress

August 28‐September 1, 2027

Amsterdam, the Netherlands

**ILAE WEBINARS**

Rassmusen Encephalitis: A Case Series on Epilepsia Partialis Continua in a LMIC

October 7, 2025

How to manage ketogenic diet therapies for refractory status epilepticus

October 16, 2025

How to use AI in clinical EEG interpretation

October 23, 2025

ILAE e‐Forum: The role of digital technologies in the management of epilepsy

November 4, 2025

How to interpret genetic data in *SCN1A*‐related disorders

November 27, 2025

ILAE e‐Forum: EEG: When, why, and how?

December 12, 2025

**OTHER CONGRESSES**

58th Annual Meeting of the Japan Epilepsy Society

October 2–4, 2025

Utsunomiya, Japan

Defining the Landscape of Late‐Onset Unexplained Epilepsy

October 15–17, 2025

South Casco, Maine, USA

Paediatric Epilepsy Training 1 ‐ 22 October 2025

22 October 2025

3rd Annual Conference of the African Neurophysiological Society

October 23–25, 2025

Accra, Ghana

2025 CLAE Annual Scientific Meeting

October 24–26, 2025

Calgary, Alberta, Canada

Epilepsy Society of Australia 39th Annual Scientific Meeting

November 5–7, 2025

Perth, Australia

3rd Advanced Course on the Pharmacological Treatment of Drug Resistant Epilepsies

November 7–9, 2025

Palma de Mallorca, Spain

42nd International Conference on Advances in Psychiatry and Mental Health

November 10–11, 2025

Dubai, UAE

In Search of Lost Time 6

November 12–14, 2025

Rome, Italy

Paediatric Epilepsy Training 2 ‐ Newcastle

13 ‐ 14 November 2025

Newcastle, UK

Paediatric Epilepsy Training 3 ‐ Newcastle

13 ‐ 14 November 2025

Newcastle, UK

Paediatric Epilepsy Training 1 ‐ 21 November 2025

21 November 2025

19th Asian Epilepsy Surgery Congress

November 21–23, 2025

Pak Shek Kok, Hong Kong SAR

The 2025 ILAE British Branch Clinical Epilepsy 1‐Day Course for Doctors in Training and Others

November 22, 2025

Birmingham, UK

Pituitary Surgery Course 2025

November 25, 2025

London, UK

5th Meeting of the British Neuroendoscopy Society

November 28, 2025

London, UK

International Neuroscience Conference 2025

29 ‐ 30 November 2025

Tbilisi, Georgia

Encephalitis 2025

December 3–4, 2025

London, UK and online

British Neurosurgical Research Group Meeting 2025

December 8–9, 2025

London, England

4th Advanced Imaging in Epilepsy

12 ‐ 14 December 2025

Rome, Italy

**2026**

Paediatric Epilepsy Training 2 ‐ Bristol

26 ‐ 27 February 2026

Bristol, UK

Paediatric Epilepsy Training 3 ‐ Bristol

26 ‐ 27 February 2026

Bristol, UK

4th International Artificial Intelligence in Epilepsy and Neurological Disorders Conference

March 16–19, 2026

San Juan, Puerto Rico

18th Eilat Conference on New Antiepileptic Drugs and Devices

May 3–6, 2026

Madrid, Spain

64th Annual Meeting of the German Society for Epileptology

June 10–13, 2026

Würzburg, Germany

1st Swiss Neuro Week

November 18–20, 2026

Bern, Switzerland

